# Hypothalamus-pituitary-adrenal axis involves in anti-viral ability through regulation of immune response in piglets infected by highly pathogenic porcine reproductive and respiratory syndrome virus

**DOI:** 10.1186/s12917-018-1414-3

**Published:** 2018-03-14

**Authors:** Jie Tong, Ying Yu, Linlin Zheng, Chong Zhang, Yabin Tu, Yonggang Liu, Jianan Wu, Hai Li, Shujie Wang, Chenggang Jiang, En-Min Zhou, Gang Wang, Xuehui Cai

**Affiliations:** 1 State Key Laboratory of Veterinary Biotechnology, Harbin Veterinary Research Institute of Chinese Academy of Agriculture Science, Harbin, 150001 People’s Republic of China; 2 Department of Preventive Veterinary Medicine, College of Veterinary Medicine, Northwest A&F University, Yangling, Shaanxi 712100 People’s Republic of China

**Keywords:** HP-PRRSV, Hypothalamus-pituitary-adrenal axis, Proinflammatory cytokines

## Abstract

**Background:**

The highly pathogenic porcine reproductive and respiratory syndrome virus (HP-PRRSV) has been responsible for several viral attacks in the Asian porcine industry, since the first outbreak in China in 2006. During the early stages of the HP-PRRSV infection, high levels of proinflammatory cytokines are noted in the host peripheral blood, which are accompanied by severe lesions in the lungs and immune system organs; these are considered as the greatest contributors to the overall disease burden. We hypothesized that the anti-PRRSV response in piglets might be mediated by the hypothalamus-pituitary-adrenal (HPA) axis, which led to a decrease in the psycho-neuroendocrinological manifestation of HP-PRRSV etiology via immune response regulation.

**Results:**

We investigated the regulation of the HPA axis in HP-PRRSV-infected piglets that were treated with 1 mg/kg body weight (b. w.)/day mifepristone (RU486) or 2 mg/kg b.w./day dexamethasone (DEX). Both RU486 and DEX enhanced the disease status of the piglets infected by the HP-PRRSV HuN4 strain, resulting in high mortality and more severe pathological changes in the lungs.

**Conclusions:**

HP-PRRSV infection activates the HPA axis, and artificial regulation of the immune-endocrine system enhances disease severity in HP-PRRSV-infected piglets. Thus, DEX and RU486 should be avoided in the clinical treatment of HP-PRRS.

**Electronic supplementary material:**

The online version of this article (10.1186/s12917-018-1414-3) contains supplementary material, which is available to authorized users.

## Background

Porcine reproductive and respiratory syndrome (PRRS) is caused by PRRS virus (PRRSV) [1]. Since the first outbreak in 2006, HP-PRRS has been the leading cause of serious economic losses in the pig industry in China [2–4]. It is generally believed that PRRSV induces lesions in immune organs in piglets, causing severe disorders of the host immune response [5–8].

Apart from the direct effect of the virus infection, the inefficient immune response may also be caused by an unbalanced neuro-endocrino-immunological (NEI) status after PRRSV infection. In this case, the hypothalamus-pituitary-adrenal (HPA) axis is considered to play a crucial role as it changes the host’s susceptibility to some infectious diseases. Under stressful conditions, such as during a robust proinflammatory response in the early stage of virus infection, the HPA axis may be triggered by high levels of proinflammatory cytokines, particularly IL-1β, IL-6, and TNF-α [9]. It has been found that the plasma levels of these cytokines increased after HP-PRRSV infection [10], which may possibly activate the HPA axis after infection. Following the activation of the whole HPA neuroendocrine-pathway, glucocorticoids (GCs) secreted by the adrenal glands are the final effector molecules that suppress violent inflammations and balance the immune response [11]. The anti-inflammatory effects of GCs are mediated via the glucocorticoid receptor (GR) [9, 12], which is a member of the steroid hormone-receptor family of ligand-dependent transcription factors (NR3C1). Accordingly, pharmacologic GCs such as dexamethasone (DEX) have been used as potential therapeutic options for virus-induced autoimmune disease and inflammations, including the Kilham rat virus (KRV) and severe acute respiratory syndromes (SARS) [13, 14]. However, the administration of excess DEX may impede the secretions of ACTH and cortisol through negative feedback loops of the HPA axis, which may also lead to other problems [15]. Hence, DEX treatment has multiple effects on the immune response in the clinical setting. In some other cases, excess GCs induce the apoptosis of lymphocyte cells, which are responsible for immune suppression during viral infection. Apharmacy GC inhibitor, mifepristone (RU486), has been used for GR blockade, and the treatment is termed as a temporary adrenalectomy. However, reports on the interactions between HP-PRRSV infection and HPA axis regulation are currently rare.

Our previous studies showed that HP-PRRSV infection consistently induced high levels of proinflammatory cytokines in the peripheral blood of infected piglets, which resulted in long-lasting high body temperatures and considerably higher mortality as compared to that induced by classical PRRSV infection. Interestingly, most of the infected piglets displayed severe thymus atrophy and lymphocyte apoptosis from 3 to 10 days after the virus infection [7, 10, 16–18]. These symptoms may develop under NEI deficient conditions, which suggests that some neuro-endocrine hormones released by the HPA axis failed to modulate the anti-viral innate immune response during the viral infection [19–21]. Thus, we hypothesized that the HPA axis was affected by the HP-PRRSV infection, which then failed to sustain homeostasis in the infected piglets. In this study, we investigated the functions of the HPA axis in HP-PRRSV-infected piglets by assessing the effects of daily treatment with 1 mg/kg body weight (b.w.) RU486 or 2 mg/kg b. w. DEX. At 10 days post infection, higher mortality and more severe pathological changes in the lungs were observed in pharmacy-treated animals, which indicate that disorders of the HPA axis enhance the disease status of piglets infected by the HP-PRRSV HuN4 strain. Furthermore, significant changes in the proinflammatory cytokine levels in peripheral blood were observed in both DEX- and RU486-treated piglets, which was consistent with our hypothesis of incomplete HPA axis function. These results provide new insights into the NEI pathophysiology associated with HP-PRRSV infection.

## Methods

### Virus

The HP-PRRSV HuN4 strain (GenBank accession no. EF635006) was used in this study as a viral inoculum after three passages through porcine alveolar macrophages (PAMs, which were isolated using a lung lavage technique from 4 to 6-week-old specific-pathogen-free piglets that were free of PRRSV, PCV_2_, CSFV, PPV, PRV, swine influenza virus, and Mycoplasma hyopneumoniae infections as previously described [22]) with a titer of 10^5.0^ TCID_50_/mL [23].

### Ethics statement

The animal experiment protocols were approved by the Animal Ethics Committee of Harbin Veterinary Research Institute of the Chinese Academy of Agricultural Sciences (CAAS), and the methods were conducted in accordance with the approved animal ethics guidelines. The Animal Ethics Committee approval number was SYXK (Hei) 2,015,042.

### Experimental design

20 PRRSV-negative piglets (Landrace× Yorkshire× crossbred, 12 males, weighting 7.5 to 9 kg and aged 28 days) were purchased from a local PRRSV free farm (Xinli farm), randomly divided into four groups (5 piglets/group) and maintained separately in isolated rooms. After 1 week of acclimation, Groups A, B and C were inoculated intranasally (i.n., without anesthesia) with 3 mL of HP-PRRSV HuN4 (10^5.5^ TCID_50_ in 3 mL DMEM medium), and Group D were sham-inoculated with 3 mL of DMEM medium. Group A was injected intramuscularly (i.m.) with DEX (Dexamethasone Sodium Phosphate Injection solution, Sanchine Pharmaceutical Co., China) at 2 mg/kg b. w. daily during the HuN4 infection. Group B was injected with RU486 (mifepristone; Sigma-Aldrich, dissolved in 50% ethanol) intra-peritoneally at 1 mg/kg b. w. within 1 day prior to the viral infection. Blood samples collected at 0 (before inoculation), 3, 5, 7 and 10 days after inoculation (days post-inoculation; DPI) were used for virological analysis. Some piglets died without euthanasia during the experimental intervention, and the other piglets were anesthetized by barbiturate overdose (intravenously), bled and euthanized humanely at 14 DPI.

### Clinical observation

Piglets were monitored daily for clinical signs including anorexia, lethargy, fever, and emaciation prior to feeding, including anorexia, lethargy, fever, and emaciation.

### Serum cytokine levels

Serum samples were collected at the designated DPI. Cytokine concentrations were measured in duplicate using commercial ELISA kits (Cloud Clone Corp, USA) according to the manufacturer’s instructions. The serum concentrations of cytokines (pg/mL) were calculated according to the recombinant standards supplied in the kits.

### Quantitative real-time PCR for detection of viral loads

Viral loads in peripheral blood were detected by TaqMan fluorescent quantitative RT-PCR (RT-qPCR) as described previously [10].

### Necropsy

The macroscopic lesions in the lungs were recorded at necropsy. Sections of the thymus and lungs were fixed in 10% neutrally-buffered formalin for histological examination using hematoxylin and eosin staining.

### Statistical analysis

No formal calculation of sample size was done due to the characteristics of the experiment. It was expected that more than three piglets per group would allow obtaining appropriate results data.

Statistical analysis was performed using GraphPad Prism software (version 5.02 for Windows; GraphPad Software). Comparisons between groups were analyzed using the analysis of variance (ANOVA) test. A *P*-value less than 0.05 was considered to indicate statistical significance.

## Results

### Disease status of piglets infected by HP-PRRSV was enhanced after treatment with RU486 or DEX

**Fig. 1:**
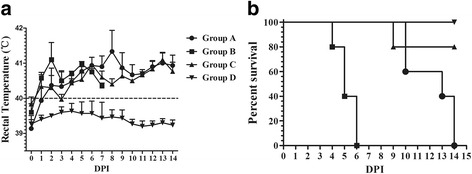
Rectal temperature and percentage survival in piglets after HuN4 infection. Daily average rectal temperatures in the four groups (**a**), each point represents the mean values (±S.D.) generated from all pigs at each DPI; and percentage survival of piglets from the four groups (**b**)

Group A (2 mg/kg b. w. DEX) maintained a high fever (40–42 °C) from 2 DPI (Fig. 1a) and showed symptoms of severe HP-PRRS disease, reaching 100% mortality at 14 DPI (Fig. 1b). Similar results were observed in Group B (RU486 1 mg/kg b. w.), reaching 100% mortality at 7 DPI (Fig. 1b). Group C showed symptoms typical of HP-PRRS infection including high fever and one piglet died as a result of HP-PRRS infection at 9 DPI. During the experiment, Groups A and Group B exhibited more severe disease than Group C, which was infected with HuN4 alone. Group D showed no symptoms during the experiment.

### Visual and histological observations

**Fig. 2:**
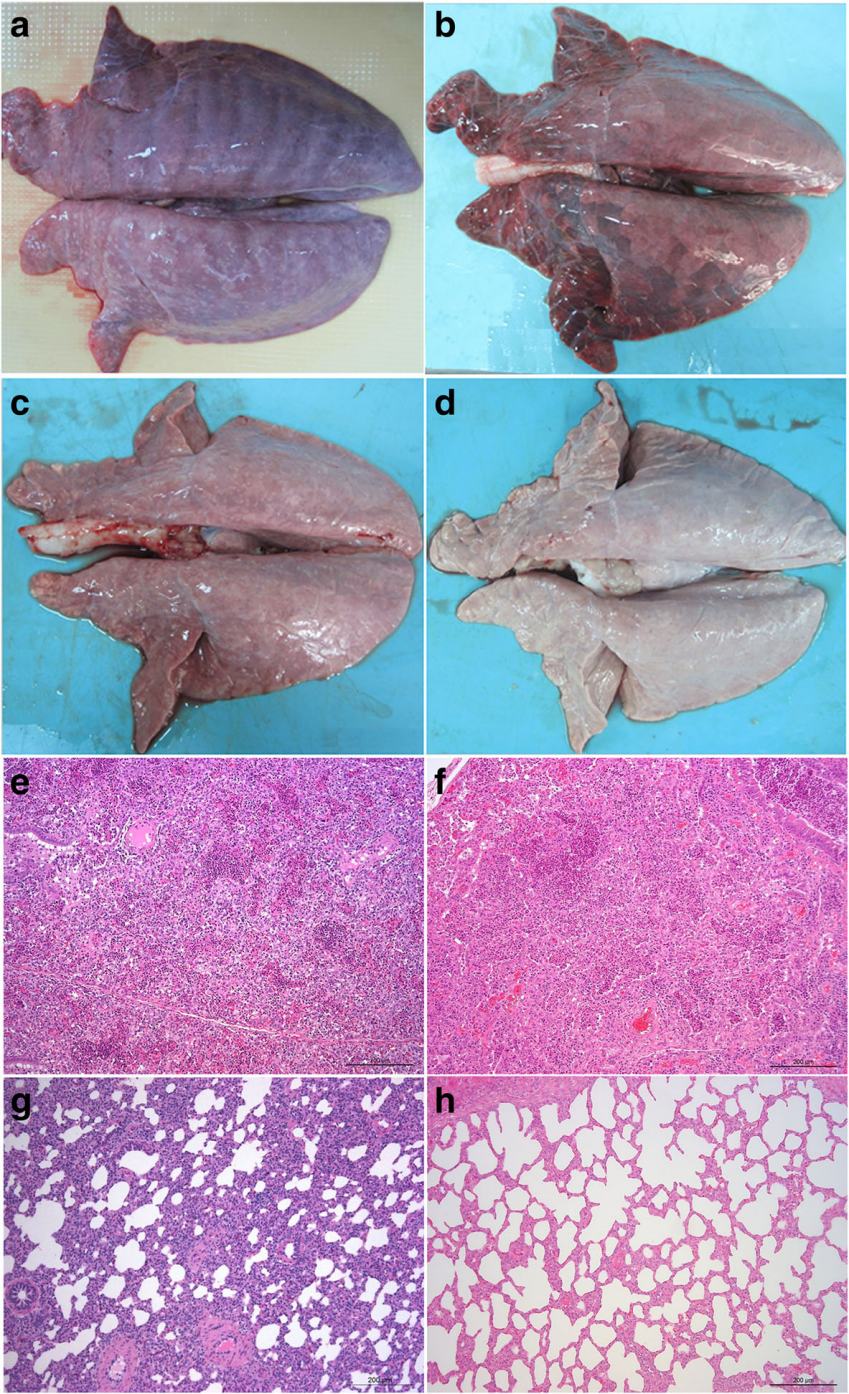
Representative images showing the gross pathological findings as well as the pathological changes in the lung of piglets from the four groups. Lung of one piglet that died in group A following HP-PRRS infection at 14 DPI (**a** and **e**), in group (**b**) at 6 DPI (**b** and **f**), humanely euthanized from Groups (**c**) (**c** and **g**) and (**d**) (**d** and **h**) at 14 DPI. **e**, **f**, **g** and **h** magnification: 100 ×

The macroscopic lesions of the lungs were recorded at necropsy. The mortality in Groups A and Group B reached 100%, and severe interstitial pneumonia accompanied by edema and congestion was observed in the lungs of these piglets (Fig. 2a and b). Observation of histopathological changes revealed suppurative pneumonia characterized by severe diffuse interstitial proliferation of small lymphocytes (Fig. 2e and f). The lungs of Group C piglets infected with HuN4 alone showed typical signs of interstitial pneumonia (Fig. 2c and g), while the lungs in Groups D piglets were normal (Fig. 2d and h).

### Viremia

Group A piglets were PRRSV-positive at 3 DPI (mean titer, 10^5.69^ copies/mL), and peaked at 10 DPI (10^8.8^ copies/mL). Group B piglets were also PRRSV-positive at 3 DPI (10^5.13^copies/mL), and peaked at 6 DPI (10^9.86^ copies/mL). The viral load in sera of Group C was 10^5.14^copies/mL at 3 DPI and peaked at 10 DPI (10^7.31^copies/mL) (Table 1).

**Table 1:** Detection of viremia and amounts of virus at different DPIs

DPI	Group A		Group B		Group C	
	Viremia^a^	PRRSV amount^b^	Viremia	PRRSV amount	Viremia	PRRSV amount
0	0/5	0	0/5	0	5/5	0
3	5/5	10^5.69 ± 0.2^	5/5	10^5.13 ± 0.2^	5/5	10^5.14 ± 0.1^
6	5/5	10^7.65 ± 0.2^	2/2^d^	10^9.86 ± 0.4^	5/5	10^6.64 ± 0.4^
10	3/3^c^	10^8.8 ± 0.3^	–	–	4/4^e^	10^7.31 ± 0.3^

^a^Viremia was detected by TaqMan fluorescent quantitative PCR (Liu al., 2010);

^b^ Each number represents the average amount of virus (copies/mL) generated from the blood of all PRRSV-positive piglets at each DPI;

^c^ Two piglets died from HP-PRRS at 10 DPI;

^d^ One piglet died from HP-PRRS at 4 DPI and two piglets died from PRRS at 5 DPI;

^e^One piglet died from HP-PRRS at 9 DPI

### Dysregulation of the HPA axis altered the proinflammatory levels in the peripheral blood of HP-PRRSV-infected piglets

**Fig. 3:**
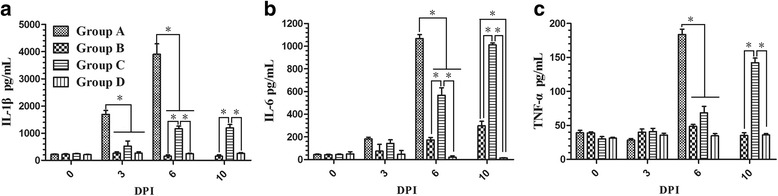
Levels of proinflammatory cytokines in the peripheral blood of piglets from the four groups. The levels of IL-1β (**a**) IL-6 (**b**) and TNF-α (**c**) were measured using commercial ELISA kits. Each point represents the mean values (±S.D.) generated from all piglets at each DPI. *: Values were significantly different (*P* < 0.05)

The concentrations of the proinflammatory cytokines IL-1β, IL-6 and TNF-α were determined for all serum samples (Fig. 3). The concentrations of IL-1β, IL-6 and TNF-α in Group A began to rise at 3 DPI, and peaked at 6 DPI. No significant changes in the serum concentrations of IL-1β, IL-6 and TNF-α were detected in Group B during the experiment. The concentrations of IL-1β, IL-6 and TNF-α in Group C also began to increase at 3 DPI, although the levels were significantly lower than those of Group A at 6 DPI.

## Discussion

Our current animal experiment was based on our previous result that HP-PRRSV infection activated the HPA axis by changing the peripheral blood hormone levels (Additional file 1: Figure S1). The present study showed that both RU486 and DEX enhanced disease in piglets infected with HuN4, with higher mortality and more severe pathological changes in the lungs of infected piglets.

Previous study has shown that low dose of DEX (1.5 mg/kg b.w.) therapy attenuates pulmonary injury and chronic lung disease in premature animals by improving pulmonary compliance [24]. However, after viral infection, for example PCV2, even such low dose of DEX treatment may accelerate the presence of post-weaning multisystemic wasting syndrome [25]. Similar to the case of PCV2, our results showed that daily treatment of DEX also suppresses the cell-mediated immunity of piglets after HP-PRRSV infection (Fig. 3). RU486 are generally characterized as progesterone and glucocorticoid receptor antagonist, and because of this activity, high dose (more than 100 mg) treatment of RU486 are commonly used as a contraceptive [26]. RU486 rarely has detrimental effects on the immune response of animals. However in our case, when the piglets were pre-infected by HP-PRRSV, the additionally treatment of RU486 significantly accelerates the development and severity of the disease. As indicated in the survival rates analysis, piglets in Group B died very early after the infection (Fig. 1b). Given the evidence of much higher levels of proinflammatory cytokines in the peripheral blood and sever lung lesions, the lethal outcome may due to the multisystemic wasting syndrome induced by severe inflammations. The uncontrolled inflammations may be related to insufficient HPA regulation that induced by RU486 treatment.

Although some studies have shown that the secretion of proinflammatory cytokines were involved in the activation of the NF-κB pathway after PRRSV infection [27, 28], our results indicated that it may also influenced by HPA axis regulation. The HPA axis is generally activated as the first line of defense against viral infection in humans [29]. GCs secreted by the adrenal glands after HPA axis activation have been found to suppress immune functions through negative feedback control of the immune system [30, 31]. Predisposition to inflammatory or autoimmune disease can cause insufficient GCs responses; however, chronic stress, predisposition to infectious diseases and other sequelae of immunosuppression can induce excessive GCs production [11, 32]. Although many types of immune-mediated diseases lead to the dysregulation of the HPA axis and impropriate GC production, GC analogues, such as DEX, have long been used as effective anti-viral agents in both humans and animals [33].

In the present study, RU486 and DEX were used to regulate the function of the HPA axis in order to improve the health status of piglets during HP-PRRSV infection. Surprisingly, neither RU486 nor DEX improved the health status of virus-infected piglets, but instead, enhanced the disease. After evaluating the proinflammatory cytokine levels in infected piglets, we found that RU486 exerted a negative effect on the host anti-inflammatory response during HP-PRRSV infection by suppressing the physiological concentrations of GCs, which play a vital role in the anti-viral process. In contrast, DEX inhibited the inflammation induced by HP-PRRSV, although the levels of proinflammatory cytokines in the peripheral blood were significantly decreased and long-lasting high fever and lung lesions were also observed in these HuN4-infected piglets. Despite the solvent of RU486 contained 50% ethanol (30 mg/ml), the dose used for each piglet was no more than 0.1 g/kg b.w., which has been demonstrated to have neither significant effect on the plasma cortisol levels nor anti-viral cell-mediate immune response, as well as no perceptible depression of the central nervous system [22, 25]. Thus, we believe that DEX induces immunosuppression during HP-PRRSV infection, allowing enhanced viral replication and leading to high mortality. These results demonstrate that both RU486 and DEX influence the anti-inflammatory responses in HP-PRRSV -infected piglets, leading to more severe disease.

## Conclusions

Our study demonstrates that HP-PRRSV infection activates the HPA axis, while artificial regulation of this immune-endocrine system enhanced the severity of disease in HP-PRRSV-infected piglets. These results indicate that DEX and RU486 should be avoided in the clinical therapy of HP-PRRS.

## Additional file

Additional file 1: **Figure S1.** Levels of Cortisol, CRH and ACTH in serum. The levels of Cortisol (A), CRH (B) and ACTH (C) were measured by the commercial ELISA kits (Cloud Clone Corp, USA). Each point represents the mean values (±S.D.) generated from all pigs on each DPI, *P < 0.05. (TIFF 383 kb)

## Abbreviations

b.w.: Body weight
DEX: Dexamethasone
DPI: days post-inoculation
GCs: Glucocorticoids
GR: Glucocorticoid receptor
HPA: Hypothalamus-pituitary-adrenal
HP-PRRSV: Highly pathogenic PRRSV
i.m.: Intramuscularly
PRRS: Porcine reproductive and respiratory syndrome
PRRSV: Porcine reproductive and respiratory syndrome virus
RU486: Mifepristone
