# Supplementary figures and images for: Hypothalamus-pituitary-adrenal axis involves in anti-viral ability through regulation of immune response in piglets infected by highly pathogenic porcine reproductive and respiratory syndrome virus
Source: BMC Vet Res. 2018 Mar 14;14:92. doi: 10.1186/s12917-018-1414-3 (PMC5853143; doi:10.1186/s12917-018-1414-3)

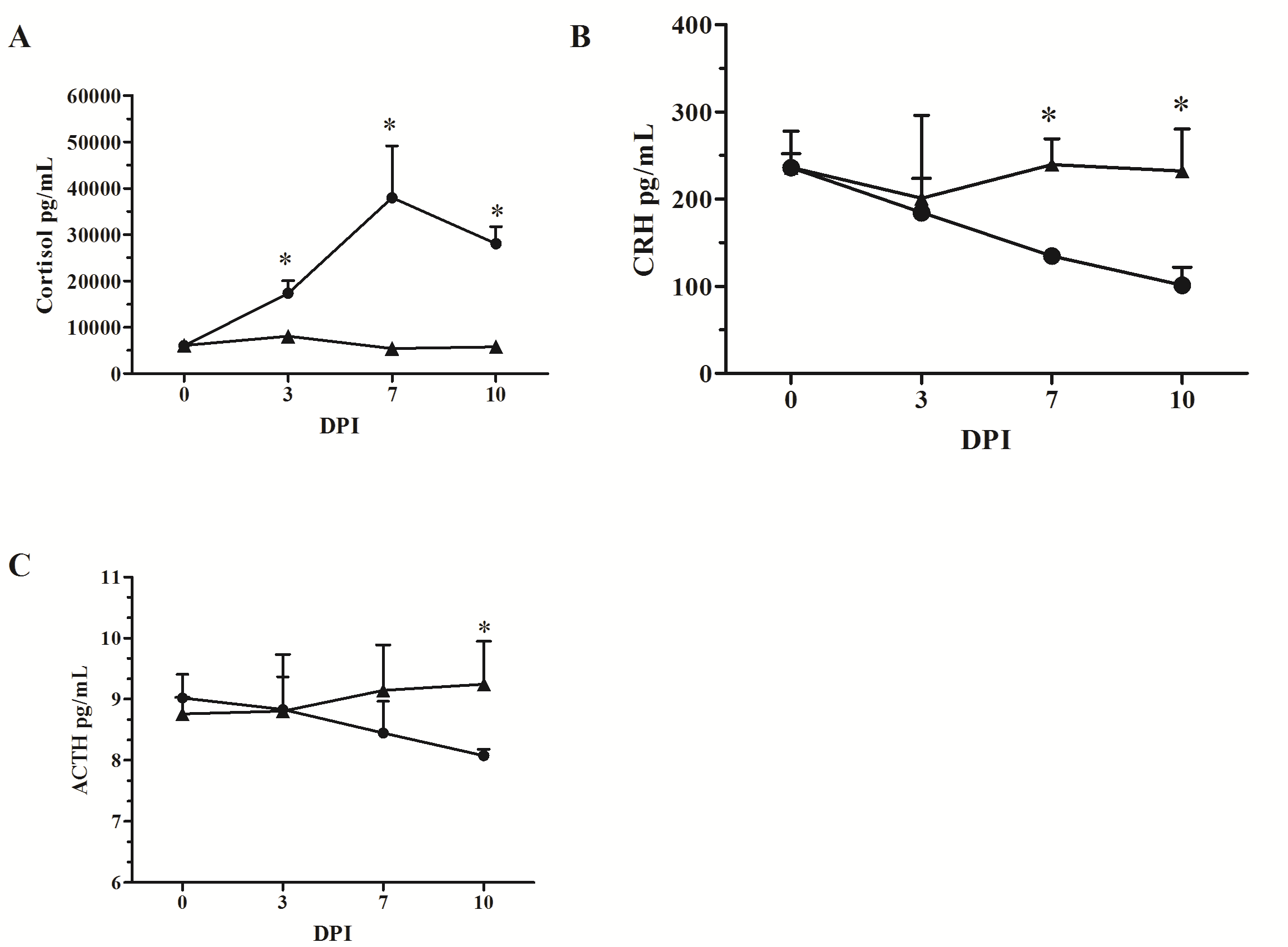

Supplement: Supplementary file 1 — Figure S1. Levels of Cortisol, CRH and ACTH in serum. The levels of Cortisol (A), CRH (B) and ACTH (C) were measured by the commercial ELISA kits (Cloud Clone Corp, USA). Each point represents the mean values (±S.D.) generated from all pigs on each DPI, *P < 0.05. (TIFF 383 kb) [file 12917_2018_1414_MOESM1_ESM.tif]
